# Effect of feeding wheat middlings and calcium lignosulfonate as pellet binders on pellet quality growth performance and lipid peroxidation in broiler chickens

**DOI:** 10.1002/vms3.344

**Published:** 2020-08-19

**Authors:** Ahmed A. Saleh, Ayman M. Elnagar, Yahya Z. Eid, Tarek A. Ebeid, Khairy A. Amber

**Affiliations:** ^1^ Department of Poultry Production Faculty of Agriculture Kafrelsheikh University Kafrelsheikh Egypt; ^2^ Department of Animal Production and Breeding College of Agriculture and Veterinary Medicine Qassim University Buraydah Saudi Arabia

**Keywords:** calcium lignosulfonate, growth performance, lipid peroxidation, pellet quality, wheat middlings

## Abstract

Basal diets supplemented with 4 kg Ca‐LS/ton of diet. Pellet quality characteristics (per cent fines, the present study was conducted to evaluate the influence of wheat middlings (WM) and calcium lignosulfonate (Ca‐LS) as pellet binders on the pellet quality characteristics, growth performance, blood parameters, nutrients digestibilities, lipid peroxidation and muscle fatty acids profile in Egyptian broiler strain. A total of 3,120 broiler chicks at 1‐day of age were divided randomly into three experimental treatments with eight replicates (130 each). The first treatment was fed the basal pelleted diets without any additives, the second treatment was fed diets including 50 kg WM/ton of diet and the third treatment was fed per cent pellets, and pellet durability index) were significantly improved in WM and Ca‐LS treatments compared with the control. Body weight gain was significantly increased, while feed intake was significantly decreased resulting in improving of feed conversion ratio significantly in WM group in comparison with control and Ca‐LS groups (*p* < .05). Nutrients apparent digestibility (dry matter, crude protein and crude fibre) were significantly improved by inclusion of WM compared with control and Ca‐LS. Plasma total cholesterol, and uric acid concentrations were significantly decreased by dietary WM in comparison with control and Ca‐LS experimental groups. Furthermore, linoleic, alpha*‐*linolenic and arachidonic acids contents in breast muscle were significantly increased by WM and Ca‐LS, while, muscle malondialdehyde concentration was significantly decreased. It could be concluded that inclusion of WM and Ca‐LS can improve pellet quality characteristics, and WM (at a level of 50 kg/ton) had positive effects on growth performance, nutrients digestibilities, lipid peroxidation and fatty acids profile in Egyptian broiler strain.

## Graphical abstract

Nowadays, with the improvements in broiler production, several attempts are done to enhance the several factors influencing the growth rate during the fatting period to attain maximum weight gain and feed efficiency. Improving formulations and the quality of the diet are among the main strategies to achieve better performance. Offering pelleted feed to poultry enhanced the economics of production via enhancing the feed consumption, feed conversion ratio (FCR) and live weight gain in broilers.

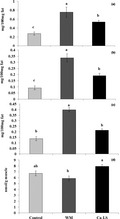

## 1 INTRODUCTION

Nowadays, with the improvements in broiler production there are several factors influencing the growth rate and feed conversion rates. Improving formulations and the quality of the diet are among the main strategies to achieve better performance (Amerah & Gracia, 2011). Offering pelleted feed to poultry enhanced the economics of production via enhancing the feed consumption, feed conversion ratio (FCR) and live weight gain in broilers (Behnke & Beyer, 2002). These enhancements improved feed intake, minimized feed loss, bigger bulk and nutrient intensity, no selective feeding, reduced time and energy expended in eating, minimized ingredient separation, extermination of feed‐borne pathogens, inactivation of enzyme inhibitors and thermal modulation of starch and protein (Behnke, 1994). Pellet quality and strength are very substantial to get the benefits connected with pelleting (Jensen, 2000). Previous researches showed that FCR of poultry was decreased as pellet fines was increased and they illustrated that dietary low quality pellets could decline some of the advantages of pelleting, but separating pellet fines via sieving is not guaranteed in broilers (Proudfoot & DeWitt, 1976; Schell & Van Heugten, 1998).

Calcium lignosulfonates (Ca‐LS) are by‐products of the paper manufacture and are utilized as pellet binders. Chemical structure of Ca‐LS is calcium 3‐(2‐hydroxy‐3‐methoxyphenyl)‐2‐[2‐methoxy‐4‐(3‐sulfonatopropyl)] phenoxy propane‐1‐sulfonate (Yang, Qiu, Pang, & Zhou, 2008). The Ca‐LS are good source of calcium (5%), sodium (0.17%) and ammonium (0.026%) salts as well as have high proportions of several wood sugars (25%–30%) and hemicelluloses (55%) (Association of American Feed Control Officials Incorporation, 1989). Lignosulfonates were utilized in poultry feeds as an effective binder when used at a level of 1%–3% (Anonymous, 1983; MacMahon, 1984). The lignosulfonate products had several utilities including dispersing, binding, complexing and emulsifying properties leading to prevent aggregation of small particles and droplets illustrated that inclusion of Ca‐LS improved pellet quality, broiler performance and nutrients digestibility (Cecilia, Toledo, & Kuznesof, 2008; Corey, Wamsley, Winowiski, & Moritz, 2014).

Wheat middlings (WM) are by‐products of the wheat milling manufacturing and do not contend with humans as food origin. The WM has the efficiency to diminish the feeding expenditures in poultry and livestock. In wheat milling process, the flour consists of 70%–75% of the grain and the remaining 25%–30% is wheat by‐products which might be used in poultry and livestock feeding (Laudadio & Tufarelli, 2012). These by‐products generally are called WM, mill feed (MF) or wheat mill run (WMR) with little consideration for the various mill process that influenced the final composition of these by‐products. The nutrient composition values of WM are 16% CP, 2,540 Kcal ME/kg, 2.7% CF, 3.5% EE, 0.55% Lysine and 0.25% Methionine (Ahmadi & Amini, 2014; Ahmadi & Tahir, 2010; Tufarelli, Khan, & Laudadio, 2011). The improving in feed pellet quality by WM might be due to two reasons, the first by variety of low‐digested materials called non‐starch polysaccharides (including cellulose, arabinoxylans, hemicelluloses, lignin made up approximately 14, 44, 19 and 1.8% DM basis; respectively) (Annison & Choct, 1991; Tufarelli & Laudadio, 2011) and, the second reason is by starch gelatinization process (Zimonja, Stevnebø, & Svihus, 2007). Classen and Bedford (1991) reported that growth performance of broiler was improved by feeding 4% of WM. The aim of the present study was to evaluate the beneficial effects of inclusion of WM and Ca‐LS as pellet binders on the pellet quality characteristics, growth performance, blood parameters, nutrients digestibilities, lipid peroxidation and muscle fatty acids profile in Egyptian broiler strain.

## 2 MATERIALS AND METHODS

### 2.1 Birds and experimental design

A total of 3,120 1‐day old unsexed Egyptian broiler strain were divided randomly into three experimental treatments with eight replicates (130 each). All birds were fed on the same starter diet (from 1 to 10 days) then the birds were divided and fed on experimental diets (grower diets from 11 to 30 days and finisher from 31 to 49 days). The first group was served as control and fed the basal diets without any additives, the second group was fed on diets containing 50 kg WM/ton of diets (the best level we used in broilers diets without and disadvantages in the performance), and the third group was fed on diets supplemented with 4 kg Ca‐LS/ton of diets (the recommended dose by Nutrivet Misr Co.). The levels of WM and Ca‐LS used in this study were determined according to the previous studies (Ahmadi & Tahir, 2010; Classen & Bedford, 1991) and according to the recommendations of the producing companies. The formulation and the chemical composition of the experimental diets are presented in Table 1. The experiment was conducted in close system farm (Research Center of New El Sabeel Company) with 21 hr light: 3 hr dark cycle. At 1 day of age, the initial brooding temperature was 34°C and relative humidity 60% and the temperature was reduced by 2°C/wk until reaching 24°C. After brooding period, room temperature was kept at 24°C with relative humidity from 50% to 60% throughout the experimental period.

**TABLE 1:** Composition and nutrient analysis of the experimental diets

		Control		Wheat Middlings		Calcium Lignosulfonate	
Ingredients, g/kg	Starter	Grower	Finisher	Grower	Finisher	Grower	Finisher
Yellow corn	554	601	653	567	622	593	646
Soybean meal, 46%	380	328	254	295	216	329	254
Corn gluten meal, 62%	7	8	24	24	43	9	25
Soy oil	19	25	30	25	29	27	32
Limestone	10	10	9.5	10	9.5	10	9.5
Dicalcium phosphate	17.5	15.5	15	16.5	16	15.5	15
Premix^a^	3	3	3	3	3	3	3
Sodium bicarbonate	1.6	1.5	1.8	1.5	1.8	1.5	1.8
Salt	3.5	3.5	3.5	3.5	3.5	3.5	3.5
L‐Lys HCl	1	1.3	1.4	1.3	1.4	1.3	1.4
DL‐Met	2.6	2.5	1.8	2.5	1.8	2.5	1.8
L‐ Threonine	0.2	0.2	0.2	0.2	0.2	0.2	0.2
Potassium Carbonate	0.6	0.5	2.8	0.5	2.8	0.5	2.8
Wheat middlings	—	—	—	50	50	—	—
Calcium lignosulfonate	—	—	—	—	—	4	4
Nutrient composition^b^							
CP, %	22.44	20.49	18.45	20.50	18.44	20.54	18.46
ME, Kcal/kg	2,948	3,037	3,136	3,032	3,132	3,033	3,133
Ca, %	0.89	0.83	0.78	0.84	0.79	0.85	0.80
Total P, %	0.73	0.67	0.64	0.67	0.63	0.67	0.64
Na, %	0.21	0.20	0.21	0.20	0.21	0.20	0.21
CL, %	0.25	0.25	0.25	0.25	0.25	0.25	0.25
Potassium, %	0.88	0.80	0.79	0.79	0.78	0.80	0.79
Lys, %	1.37	1.25	1.06	1.25	1.05	1.25	1.06
Meth, %	0.63	0.60	0.52	0.61	0.53	0.60	0.52
Meth + Cyst, %	0.99	0.93	0.82	0.94	0.84	0.93	0.82
EE, %	4.46	5.14	5.74	5.17	5.69	5.30	5.91
CF, %	3.42	3.24	2.97	3.14	2.86	3.22	2.96

Abbreviations: CP, crude protein; ME, metabolizable energy; Ca, calcium; total P, total phosphorus; Na, sodium; CL, chloride, Lys, lysine; Meth, methionine; Meth + Cyst, methionine + cysteine; EE, ether extract; CF, crude fibre.

^a^ Each 3 kg of vit and Min in Premix contain: 6,000,000 IU vit A, 900,000 IU vit D3, 40,000 mg vit E, 2,000 mg vit K, 2,000 mg vit B1, 4,000 mg vit B2, 2,000 mg vit B6, 10 mg vit B12, 50,000 mg Niacin, 10,000 mg pantothenic acid, 50 mg Biotin, 3,000 mg Folic acid, 250,000 mg choline, 50,000 mg Zn, 8,500 mg Mn, 50,000 mg Fe, 50,000 mg Cu, 200 mg I, 100 mg Se and 100 mg Co.

^b^ According to NRC (1994).

### 2.2 Pellet processing and pellet quality

Mash feed was conditioned in a 1.3 × 0.31‐m (length by diameter), short‐term (10 s) conditioner to a temperature of 80°C, with a gauge steam pressure just before the conditioner of 262 kPa (38 psi). Conditioned mash temperature was monitored with an 80 PK‐24 temperature probe (American Society of Agricultural Engineers, 1997). The temperature probe was entered at the interface between the end of the conditioning barrel and beginning of the chute that connects to the pellet die chamber. Average of feed inserting the conditioner and conditioner shaft revolutions per minute were held constant for all treatments. Pellets were manufactured using a 38.1‐ (effective thickness) × 4.76‐mm pellet die without relief and a 40 HP California Pellet Mill, and then cooled on a horizontal belt cooler using forced ambient air. Electrical energy usage of the conditioner and pellet mill was determined during each run with a Square D amperage meter (McEllhiney, 1994). Pellet mill motor amperage was gauged by a Hobo U12 Data Logger. Pellet durability index (PDI), New Holmen Pellet Tester (NHPT), Per cent fine, Production rate, Conditioned mash temperature, Pellet mill motor amperage and Per cent pellets were measured according to the methods of (Cutlip et al., 2008).

### 2.3 Growth performance and carcass parts

Body weight was measured weekly and feed intake was determined daily during the experimental period. Dead birds were recorded daily. The corrected feed conversion ratio (FCR) was calculated. Feed consumption and FCR were adjusted for mortalities when appropriate. At 49 days, 150 birds (30/treatment) were weighted and slaughtered and then dissected to measure the carcass yield, weights of breast muscle, thigh muscle, liver and abdominal fat. Carcass yield was calculated relative to live weight prior to slaughter using the following formula: Carcass yield = [(Carcass weight/live weight) × 100].

### 2.4 Apparent nutrient digestibility

At the last 3 days of the experiment, excreta and feed were collected and weighted by 30 birds from each groups. Then, the samples were dried by the drying oven at 60°C for 24 hr. The whole dried samples were then homogenized after drying. Samples were randomly taken and ground to analysis according to (AOAC, 1980) for crude protein (CP, Method 968.06), ether extract (EE, Method 920.39) and crude fibre (CF, Method 932.09). The calculations for nutrients digestibility were as follows; nutrients digestibility (%) = (total nutrient intake−total nutrient excreted)/total nutrient intake × 100.

### 2.5 Blood samples and plasma biochemical analysis

At 49 days, blood samples (12/treatment) were gathered in heparinized test tubes and quickly centrifuged (3,000 rpm for 20 min at 5°C) to separate the plasma. Plasma was stored at −20°C until further analysis. Plasma total cholesterol, glutamic oxalacetic transaminase (GOT), glutamate pyruvate transaminase (GPT), glucose, total protein, albumin, globulin, uric acid, ceratenine and calcium were measured calorimetrically by using commercial kits (Diamond Diagnostics) according to the procedure outlined by the manufacturer.

### 2.6 Muscle biochemical analysis

The analysis of muscle fatty acids (6 samples per treatment) was carried out by gas liquid chromatography (GLC) using a Shimadzu gas chromatograph GC‐4 CM (PFE) equipped with a flame ionization detector (FID) according to the procedure of (Saleh, 2013). Concentration of muscle malondialdehyde (MDA) was determined according to (Ohkawa, Ohishi, & Yagi, 1979).

### 2.7 Statistical analysis

The differences between the treatment groups and the control group were analysed one‐way ANOVA using SPSS Statistics 17.0 (Statistical Packages for the Social Sciences, released 23 August 2008). Tukey^٫^s multiple comparison test was used to identify which treatments conditions were significantly different from each other at a significance level of *p* < .05.

## 3 RESULTS

Data presented in Table 2 explain the effect of WM and Ca‐LS on pellet quality characteristics including pellet durability index (PDI), New Holmen Pellet Tester (NHPT), per cent fines, production rate, conditional mash temperature, pellet mill motor amperage and per cent pellets. The PDI, and NHPT per cent pellets were significantly increased in WM and Ca‐LS treatments compared with control treatment. However, per cent fines was significantly reduced (*p* < .05). While, production rate was significantly increased (*p* < .05) by addition of WM compared with control and Ca‐LS treatments, however, pellet mill motor amperage was significantly decreased. Conditional mash temperature was not significantly influenced by treatments.

**TABLE 2:** Effect of using wheat middlings (WM) and calcium lignosulfonate (Ca‐LS) as pellet binders on pellet quality

	Control	WM	Ca‐LS
PDI, %	90.3^b^ ± 0.88	93^ab^ ± 0.58	94.67^a^ ± 0.88a
NHPT, %	75.33^b^ ± 0.88	86.33^a^ ± 0.88	89^a^ ± 0.58
Per cent fines, %	7.5^a^ ± 0.115	4^b^ ± 0.577	4.3^b^ ± 0.173
Production rate, tone/hr	19.67^b^ ± 0.33	21.67^a^ ± 0.33	20.33^b^ ± 0.33
Conditioned mash temperature, °C	76.5 ± 0.265	77 ± 0.577	75.667 ± 1.202
Pellet mill motor amperage	103.67^a^ ± 0.882	97.67^b^ ± 0.882	103.33^a^ ± 1.202
Per cent pellets, %	92.5^b^ ± 0.12	96^a^ ± 0.57	95.7^a^ ± 0.17

Note

Values are expressed as means ± standard error. Data were analysed by one‐way analysis of variance and Tukey's multiple tests. ^a‐c^Means within the same row with different superscripts differ (*p* < .05).

Abbreviations: PDI, Pellet durability index; NHPT, New Holmen Pellet Tester.

Effect of inclusion of WM and Ca‐LS as pellet binders on body weight gain, feed intake, FCR, carcass yield, breast muscle weight, thigh muscle weight, liver weight and abdominal fat weight were summarized in Table 3. Inclusion of WM significantly increased (*p* < .05) final body weight, body weight gain and breast muscle weight, while, abdominal fat and FCR were significantly decreased. However, there is no difference between Ca‐LS supplementation groups with the control group in body weight and feed intake and mortality rate. Whereas, carcass yield, thigh muscle and liver weights were not significantly affected by inclusion of WM and Ca‐LS.

**TABLE 3:** Effect of feeding wheat middlings (WM) and calcium lignosulfonate (Ca‐LS) as pellet binders on growth performance and carcass traits in Egyptian broiler strain

	Control	WM	Ca‐LS
Initial body weight, g, 11 days	312.15 ± 1.86	314.11 ± 0.68	313.82 ± 1.16
Final body weight, g. 49 days	1,630.53^b^ ± 6.11	1,660.74^a^ ± 5.49	1624.90^b^ ± 5.19
Body weight gain, g (11−49 days)	1,318.38^b^ ± 8.68	1,346.63^a^ ± 14.82	1,311.62^b^ ± 7.55
Feed intake, 11−49 days	3,577.8^a^ ± 24.82	3,533.3^ab^ ± 10.69	3,460.3^b^ ± 41.39
Mortality rate, % (11−49 days)	1.06 ± 0.06	0.96 ± 0.02	1.06 ± 0.03
FCR, 11−49 days	2.195^a^ ± 0.015	2.128^b^ ± 0.008	2.129^b^ ± 0.027
Carcass yield, g/100 g BW	70.6 ± 1.34	70.2 ± 1.95	69.89 ± 1.62
Breast muscle weight, g/100 g BW	19.269^b^ ± 0.214	20.698^a^ ± 0.424	19.610^ab^ ± 0.467
Thigh muscle weight, g/100 g BW	17.29 ± 0.497	18.64 ± 0.435	17.07 ± 0.642
Liver weight, g/100 g BW	1.77 ± 0.061	1.76 ± 0.085	1.60 ± 0.081
Abdominal fat weight, g/100 g BW	2.16^ab^ ± 0.134	1.92^b^ ± 0.096	2.42^a^ ± 0.131

Note

Values are expressed as means ± standard error. Data were analysed by one‐way analysis of variance and Tukey's multiple tests. ^a‐c^Means within the same row with different superscripts differ (*p* < .05).

Abbreviations: FCR, feed conversion ratio; BW, body weight.

Table 4 shows the nutrients apparent digestibility of broiler chickens fed WM and Ca‐LS. Dry matter, crude protein and crude fibre apparent digestibility were significantly improved by WM compared with control and Ca‐LS. However, calcium and phosphors digestibility were significantly improved by Ca‐LS.

**TABLE 4:** Effect of feeding wheat middlings (WM) and calcium lignosulfonate (Ca‐LS) as pellet binders on nutrients apparent digestibility in Egyptian broiler strain

	Control	WM	Ca‐LS
DMD, %	87.79^b^ ± 0.023	92.72^a^ ± 0.116	87.94^b^ ± 0.029
CPU, %	82.79^b^ ± 0.310	89.18^a^ ± 1.026	75.66^c^ ± 0.638
EEU, %	75.30 ± 0.221	75.79 ± 0.211	76.52 ± 0.530
CFU, %	72.43^a^ ± 0.169	72.43^a^ ± 0.169	65.86^b^ ± 0.516
Ca, %	60.16^c^ ± 0.079	63.86^b^ ± 0.122	75.49^a^ ± 0.104
P, %	63.95^b^ ± 0.162	58.45^c^ ± 0.394	72.47^a^ ± 0.419

Note

Values are expressed as means ± standard error. Data were analysed by one‐way analysis of variance and Tukey's multiple tests. ^a‐c^Means within the same row with different superscripts differ (*p* < .05).

Abbreviations: CPU, crude protein digestibility; DMD, dry matter digestibility; EEU, ether extract digestibility; CFU, crude fibre digestibility.

The influences of inclusion of WM and Ca‐LS on plasma concentrations of total cholesterol, glucose, GOT, GPT, total protein, albumin, globulin, ceratinine, uric acids and calcium are presented in Table 5. Inclusion of WM decreased significantly (*p* < .05) plasma level of total cholesterol and uric acid, however, plasma calcium was significantly increased in WM and Ca‐LS groups compared with control, but plasma level of GPT, GOT, total protein, globulin were not significantly affected.

**TABLE 5:** Effect of feeding wheat middlings (WM) and calcium lignosulfonate (Ca‐LS) as pellet binders on plasma parameters in Egyptian broiler strain

	Control	WM	Ca‐LS
Plasma TC, mg/dl	138.83^a^ ± 0.345	128.17^b^ ± 1.618	134.08^a^ ± 1.836
Plasma glucose, mg/dl	220.50 ± 0.485	222.92 ± 0.743	225.58 ± 0.259
Plasma GPT, (U/I)	90.25 ± 0.329	92.33 ± 0.142	95.75 ± 0.131
Plasma GOT, (U/I)	326.83 ± 0.322	330.83 ± 1.167	329.16 ± 0.423
Plasma TP, mg/dl	4.48 ± 0.066	4.51 ± 0.055	4.04 ± 0.036
Plasma globulin, mg/dl	2.61 ± 0.036	2.33 ± 0.025	2.14 ± 0.019
Plasma ceratnin, mg/dl	0.253 ± 0.004	0.259 ± 0.010	0.270 ± 0.003
Plasma uric acid, mg/dl	17.25^a^ ± 0.218	15.67^b^ ± 0.280	16.00^b^ ± 0.275
Plasma calcium, mg/dl	8.51^b^ ± 0.020	9.52^a^ ± 0.070	9.75^a^ ± 0.070

Note

Values are expressed as means ± standard error. Data were analysed by one‐way analysis of variance and Tukey's multiple tests. ^a‐c^Means within the same row with different superscripts differ (*p* < .05).

Abbreviations: GOT, glutamic oxalacetic transaminase; GPT, glutamate pyruvate transaminase; TC, total cholesterol; TP, total protein.

**FIGURE 1:**
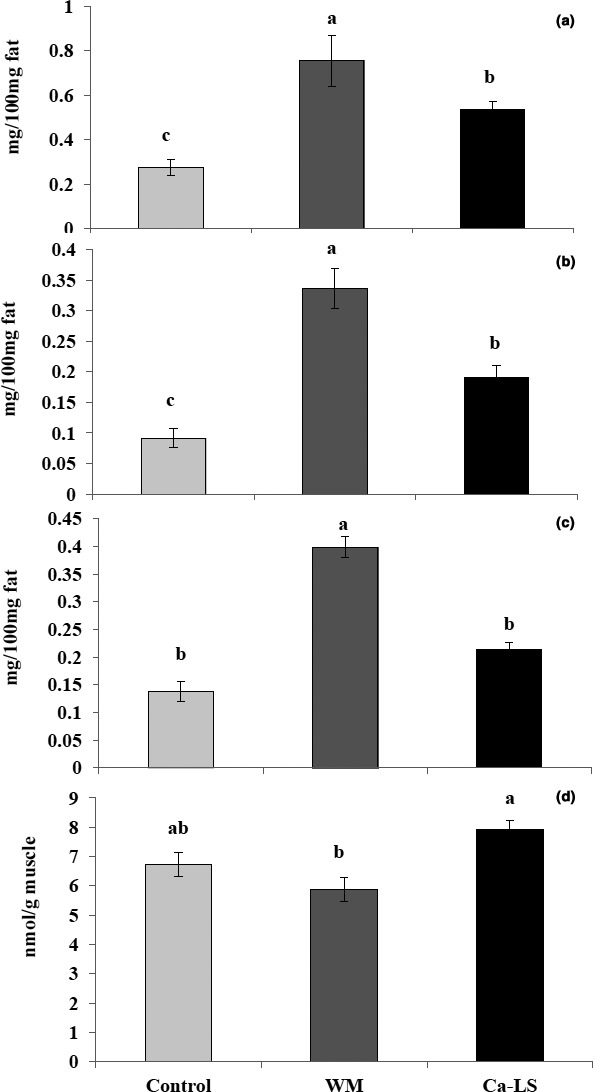
Effect of feeding wheat middlings (WM) and calcium lignosulfonate (Ca‐LS) as pellet binders on fatty acids profile and lipid preoxidation in the muscles of Egyptian broiler strain. Linoleic (a), alpha*‐*linolenic (b), arachidonic acids (c) and MDA (d). Values are means represented by vertical bar. ^a,b,c,d^Mean values with unlike letters were significantly different (*p* < .05)

Interestingly, in Figure 1, it is clear that muscle unsaturated fatty acids linoleic, alpha*‐*linolenic and arachidonic acids were significantly increased (*p* < .05) by inclusion of WM and Ca‐LS, while, MDA concentration in muscle was significantly decreased.

## 4 DISCUSSION

The levels of WM and Ca‐LS used in this study was determined according to the previous studies (Ahmadi & Tahir, 2010; Classen & Bedford, 1991) and according to the recommendations of the producing companies. Results of the present experiment proved that pellet quality characteristics were improved by using the nature pellet binders like, WM or chemical pellet binder like, Ca‐LS. These results are in accordance with different investigators (Acar, Moran, Revington, & Bilghj, 1991; Waldroup, Ritchie, & Ramsey, 1982), who found that differential sifting of feeds illustrated that inclusion of Ca‐LS improved the percentage of intact pellets by 56% (*p* < .001). Consequently, the percentage of feed kept on a 4.75‐mm screen elevated to 67.1% with Ca‐LS from 43.1% for the control. Waldroup et al. (1982) observed that the intact pellet was 72.5% when 1% Ca‐LS was used and it was reduced to 55.9% when 1.5% Ca‐LS was used. As shown in Table 2, PDI and per cent pellets were significantly increased and per cent fines were significantly decreased by inclusion of WM and Ca‐LS. These findings are in correspondence with Corey et al. (2014), who noted that inclusion of Ca‐LS increased PDI and improved MPDI significantly. The improvements in PDI and MPDI were probably connected with the transformation of Ca‐LS to be liquid by steam conditioning, filling interstitial space of feed particles, and stiffening upon drying, and consequently improved pellet quality characteristics (Ouyang, Qiu, & Chen, 2006). Additionally, the using of Ca‐LS minimized pellet temperature after extrusion significantly. Probably Ca‐LS elucidated dispersing agent qualities which let rheology of particles to be preserved throughout pellet extrusion, and reducing friction during the processing (Pure Lignin Environmental Technology, 2009). Wheat middlings contents high quantity of non‐starch polysaccharides (NSP) and highly water soluble NSP such as pentosans which might be related to the improvements in pellet quality characteristics (Gheisari, Bahadoran, & Tadayonfar, 2003; Saleh, Paray, & Dawood, 2020). Therefore, it could be mentioned that inclusion of pellet binders (WM and Ca‐LS) improved pellet quality characteristics because CaLS becomes liquid with steam conditioning, filling interstitial space of feed particles, and hardening upon drying. Also, WM had high level of cellulose and lignin, which might be involved in increasing gelatinization, leading to improving pellet quality characteristics.

Growth performance was significantly affected by inclusion of MW and Ca‐LS. As illustrated in Table 3, inclusion of WM increased body weight, and breast muscle weight, however, inclusion of Ca‐LS decreased feed intake and did not affect muscles weights. Inclusion of WM and Ca‐LS improved FCR compared with control treatment. Kivimae (1978) illustrated that feed efficiency was not significantly affected by inclusion of lignosulfonates in broilers and laying hens. Also, 6% Ca‐LS had a significant effect on loose droppings. Proudfoot and DeWitt (1976) stated that pellets including 2.5% Ca‐LS resulted in magnification of ceca in chickens. They also noted that inclusion of high percentages of Ca‐LS had no impact on performance of chickens. Also, Waldroup et al. (1982) observed that inclusion of Ca‐LS enhanced pellet integrity, while, feed intake and body weight gain were not influenced in broilers. Data of this study proved that abdominal fat weight was significantly increased by Ca‐LS inclusion in comparison with control treatment. These results are in harmony with Acar et al. (1991) who found that carcass yield was not influenced; while, abdominal fat was elevated approximately 4.8% (*p* < .05) by addition of Ca‐LS. Growth performance and breast muscle weights were significantly improved by feeding WM (Table 3). These findings disagree with Stapelton, Bragg, and Biely (1980) who indicated that addition of WM had no significant effect in daily weight gain, final body weight and FCR in broiler chickens. Also, Saki and Alipana (2005) showed that no significant effects were noted on feed intake, final body weight and carcass characteristics in broilers fed WM. On contrarily, Gheisari et al. (2003) reported that it is possible to use WM at 30% levels in the diet of broiler chicks without any undesirable effects on their performance. Moreover, the growth was improved by feeding 10% of WM at because the high concentration of protein and amino acids in WM and the fibre content in WM may be give good chance for producing enzymes and improved the digestibility (Gheisari et al., 2003). Thus, it might be noted that dietary MW and Ca‐LS improved growth performance significantly in broiler chickens and this might be attributed to the enhancement of pellet integrity, and magnification of ceca. Furthermore, by taking into account our results in nutrients digestibility, it seems more likely that inclusion of MW and Ca‐LS may enhance the growth performance of broiler chickens.

Crude protein and crude fibre apparent digestibilities were significantly decreased by feeding Ca‐LS compared with control and MW experimental groups. On the other hand, calcium digestibility was significantly improved by inclusion of Ca‐LS. Corey et al. (2014) reported that inclusion of Ca‐LS in a proper level of might improve nutrient digestibility. In addition to improving pellet quality, Ca‐LS supplementation might be connected with enhancing the digestibility of amino acids (Wamsley & Moritz, 2012). The Ca‐LS might be served out as a soluble fibre and combined in the broiler's ceca. Once they enter the gut they could be fermented and lowered the caecal pH from 6.69 to 6.34 in broilers, offering a prebiotic action similar to FOS (Moran & Conner, 1992; Saleh, Hayashi, Ijiri, & Ohtsuka, 2015). Moreover, the inclusion of 1.25% lignosulfonate binder reduced *Salmonella typhimurium* significantly (Moran & Bilgil, 1992). Protein and fibre digestibilities were improved by feeding WM and this might be connected with its low content of the NSP (e.g., pentosans), which consequently might be involved in reducing the viscosity of digestives and improved nutrients digestibility (Classen, 1996; Gheisari et al., 2003; Saleh, Ebeid, & Abudabos, 2018; Saleh, Ragab, Ahmed, Abudabos, & Ebeid, 2018). In Table 3, calcium digestibility was significantly improved by inclusion of WM and Ca‐LS in comparison with control treatment. These findings are in correspondence with Jaroni, Scheideler, Beck, and Wyatt (1999), who documented that calcium and phosphorus digestibility was improved by feeding WM to Leghorn hens.

Plasma total cholesterol, and uric acid were significantly decreased by inclusion of WM and Ca‐LS compared with control treatment. Moreover, muscle MDA content was decreased and unsaturated fatty acids were increased in current study. Cardiovascular diseases (CVD) became the most serious health problem in the world (World Health Organization, 2015). A reduction of plasma total cholesterol concentration is an essential for the public health to control CVD (Grundy et al., 2004). Moreover, consuming of whole grains such as oat, barley and WM reduced plasma total cholesterol which connected with reducing the CVD by 20%–25% (Jensen et al., 2006). Wheat middlings contain significant amounts of vitamins, phytochemicals, minerals and dietary fibre (Kristensen et al., 2012), and particularly viscous fibres, e.g., β‐glucan's and WM reduced cholesterol and increased unsaturated fatty acids in blood serum. Moreover, feeding of whole grain decrease the cholesterol synthesis (Saleh, Ahmed, & Ebeid, 2019; Wang, Lichtenstein, Lamon‐Fava, & Jacques, 2006). Also, WM is rich in some phytochemicals, like phenols this chemical have antioxidant effects so it may be the reason that MDA was decreased by feeding WM in this study. On the other hand, Kivimäe (1978) concluded that Ca‐LS did not affect liver and kidney function and reported that it was very safe for using as poultry feed additives. Also, Farran, Pietsch, and Chabrillat (2013) reported that feeding different sources of fibre like WM significantly lowered abdominal fat pad and decreased lipid preoxidation in broiler chickens. It might be assumed that, in the present study, pellet binders (WM and Ca‐LS) reduced muscle content of MDA and unsaturated fatty acids and these positive effects are due to their contents of vitamins and phytochemicals, which are involved in enhancing the antioxidative properties and minimizing lipid peroxidation.

Interestingly, as shown in Figure 1, unsaturated fatty acids like linoleic, alpha*‐*linolenic and arachidonic acids were significantly increased by feeding WM compared with control and Ca‐LS. Indeed, insoluble dietary fibres are used as an energy diluent in diets, meanwhile, using it in mild amounts had a positive effect in gut development and function (Mateos, Jiménez‐Moreno, Serrano, & Lázaro, 2012; Saleh, Ijiri, & Ohtsuka, 2014). Moreover, low levels of cellulose from WM has a positive effect on nutrient retention, diminished plasma cholesterol concentration, and modified lipid composition in liver and adipose tissue in broilers (Safaa, Jiménez‐Moreno, Frikha, & Mateos, 2014; Saleh, Ohtsuka, Yamamoto, & Hayashi, 2013). On contrarily, lignin suppressed pancreatic enzymes concentrations in vitro and had a powerful capability to restrict cholesterol and bile acids from a micellar solution (Jung & Fahey, 1983; Saleh, Ragab, et al., 2018), that might influence lipid absorption and fatty acids metabolism by increasing unsaturated fatty acids and decreased saturated fatty acids.

## 5 CONCLUSIONS

Based on the obtained data, it could be concluded that inclusion of 50 kg WM/ton of diet or 4 kg Ca‐LS/ton of diet improved pellet quality characteristics, and WM had positive effects on growth performance, nutrients digestibilities, lipid peroxidation and fatty acids profile in muscles of Egyptian broiler strain.

## CONFLICT OF INTEREST

The authors declare no conflict of interest.

## AUTHOR CONTRIBUTIONS

Ahmed Ali Saleh: Conceptualization; Data curation; Formal analysis; Funding acquisition; Investigation; Methodology; Project administration; Resources; Software; Supervision; Validation; Visualization; Writing‐original draft; Writing‐review & editing. Ayman elnaggar: Conceptualization; Data curation; Formal analysis; Funding acquisition; Methodology; Project administration; Resources; Software; Writing‐original draft. yahya Eid: Conceptualization; Methodology; Project administration; Supervision; Writing‐review & editing. Tarek Ebeid: Writing‐original draft; Writing‐review & editing. khairy amber: Conceptualization; Project administration; Supervision.

### PEER REVIEW

The peer review history for this article is available at https://publons.com/publon/10.1002/vms3.344.

## References

[vms3344-bib-0001] Acar, N. , Moran, E. T. , Revington, W. H. , & Bilghj, S. F. (1991). Effect of improved pellet quality from using a calcium lignosulfonate binder on performance and carcass yield of broilers reared under different marketing schemes. Poultry Science, 70, 1339–1344. 10.3382/ps.0701339

[vms3344-bib-0002] Ahmadi, K. , & Amini, B. (2014). Determination of chemical composition and suitable level of wheat middling’s in broiler diets. International Journal of Plant, Animal and Environmental Sciences, 4, 454–459.

[vms3344-bib-0003] Ahmadi, K. , & Tahir, K. (2010). A study on wheat middlings’s usage on Turkey’s performances. Australian Journal of Basic and Applied Sciences, 4, 5630–5635.

[vms3344-bib-0004] Amerah, A. M. , & Gracia, M. I. (2011). Influence of three bacillus subtilis strains combination on the performance; intestinal morphology and blood parameters of broilers fed wheat‐based diet. Atlanta, GA: IPSF.

[vms3344-bib-0005] American Society of Agricultural Engineers . (1997). Cubes, pellets, and crumbles‐Definitions and methods for determining density, durability, and moisture. Standards 1997. St. Joseph, MI: American Society of Agricultural and Biological Engineers. ASAE S269.4.

[vms3344-bib-0006] Annison, G. , & Choct, M. (1991). Anti‐nutritive activities of cereal non‐starch polysaccharides in broiler diets and strategies mini‐mizing their effects. World's Poultry Science Journal, 47, 232–242. 10.1079/WPS19910019

[vms3344-bib-0007] Anonymous (1983). Special report: Binders. Milling, 166(2), 31–33.

[vms3344-bib-0008] AOAC (1980). Official methods of analysis (13th ed.). Washington, DC: Association of Official Analytical Chemists.

[vms3344-bib-0009] Association of American Feed Control Officials Incorporation . (1989). Official Publications (pp. 189). Atlanta, GA: Association of American Feed Control Office Inc.

[vms3344-bib-0010] Behnke, K. C. (1994). Feed manufacturing technology: Current issues and challenges. Animal Feed Science and Technology, 62, 49–57. 10.1016/S0377-8401(96)01005-X

[vms3344-bib-0011] Behnke, K. C. , & Beyer, R. S. (2002). Effect of feed processing on broiler performance. VIII. International Seminar on Poultry Production and Pathology, Santiago, Chile.

[vms3344-bib-0012] Cecilia, M. , Toledo, F. , & Kuznesof, P. M. (2008). Calcium lignosulfonate chemical and technical assessment (pp. 1–8). Rome, Italy: 69th JECFA.

[vms3344-bib-0013] Classen, H. L. (1996). Cereal grain starch and exogenous enzymes in poultry diets. World's Poultry Science Journal, 62, 21–27.

[vms3344-bib-0014] Classen, H. , & Bedford, M. (1991). Use of enzymes to improve the nutritive value of poultry feeds In Garnsworthy P. C., & Wiseman J. (Eds.), Recent advances in animal nutrition (pp. 95–116). Oxford, UK: Butterwort‐Heinemann Ltd.

[vms3344-bib-0015] Corey, A. M. , Wamsley, K. G. S. , Winowiski, T. S. , & Moritz, J. S. (2014). Effects of calcium lignosulfonate, mixer‐added fat, and feed form on feed manufacture and broiler performance. Journal of Applied Poultry Research, 23, 418–428. 10.3382/japr.2013-00916

[vms3344-bib-0016] Cutlip, S. E. , Hott, J. M. , Buchanan, N. P. , Rack, A. L. , Latshaw, J. D. , & Moritz, J. S. (2008). The effect of steam conditioning practices on pellet quality and growing broiler nutritional value. Journal of Applied Poultry Research, 17, 249–261. 10.3382/japr.2007-00081

[vms3344-bib-0017] Farran, M. T. , Pietsch, M. , & Chabrillat, T. (2013). Effect of lignocellulose on the litter quality and the ready to cook carcass yield of male broilers. Actes des 10èmes Journées de Recherche Avicole et Palmipèdes à Foie Gras, 2013 Mars 26–28; La Rochelle. France. 917–921.

[vms3344-bib-0018] Gheisari, A. , Bahadoran, R. , & Tadayonfar, S. (2003). Determination of chemical composition and suitable levels of wheat feed screening and macaroni wastes in broiler chick diets. Journal of Science and Technology of Agriculture and Natural Resources, 7, 161–169.

[vms3344-bib-0019] Grundy, S. M. , Cleeman, J. I. , Merz, C. N. , Brewer, H. B. , Clark, L. T. , Hunninghake, D. B. , … Stone, N. J. (2004). Implications of recent clinical trials for the National Cholesterol Education Program Adult Treatment Panel III guidelines. Circulation, 110, 227–239. 10.1161/01.CIR.0000133317.49796.0E 15249516

[vms3344-bib-0020] Jaroni, D. , Scheideler, S. E. , Beck, M. , & Wyatt, C. (1999). The effect of dietary wheat middlings and enzyme supplementation. 1. Late egg production efficiency, egg yields and egg composition in two strains of leghorn hens. Poultry Science, 78, 841–847. 10.1093/ps/78.6.841 10438127

[vms3344-bib-0021] Jensen, L. S. (2000). Influence of pelleting on the nutritional needs of poultry. Asian Australasian Journal of Animal Sciences, 13, 35–46.

[vms3344-bib-0022] Jensen, M. K. , Koh‐Banerjee, P. , Franz, M. , Sampson, L. , Gronbaek, M. , & Rimm, E. B. (2006). Whole grains, bran, and germ in relation to homocysteine and markers of glycemic control, lipids, and inflammation. The American Journal of Clinical Nutrition, 83, 275–283. 10.1093/ajcn/83.2.275 16469984

[vms3344-bib-0023] Jung, H. G. , & Fahey, G. C. (1983). Nutritional implications of phenolic monomers and lignin: A review. Journal of Animal Science, 57, 206–219. 10.2527/jas1983.571206x

[vms3344-bib-0024] Kivimäe, A. (1978). Effects of lignosulphonate on poultry when used as a binder in compound feed. Archiv Fur Geflugelkunde, 42, 238–245.

[vms3344-bib-0025] Kristensen, M. , Toubro, S. , Jensen, M. G. , Ross, A. B. , Riboldi, G. , Petronio, M. , … Astrup, A. (2012). Whole grain compared with refined wheat decreases the percentage of body fat following a 12‐week, energy‐restricted dietary intervention in postmenopausal women. The Journal of Nutrition, 142, 710–716. 10.3945/jn.111.142315 22357746

[vms3344-bib-0056] Laudadio, V. , & Tufarelli, V. (2012). Effect of treated field pea (Pisum sativum L. cv Spirale) as substitute for soybean meal in a wheat middlings‐based diet on egg production and quality of early laying brown hens. Archiv fur Geflugelkunde, 76, 1–5.

[vms3344-bib-0026] MacMahon, M. J. (1984). Additives for physical quality of animal feed In Beaven D. A. (Ed.), Manufacture of animal feed (pp. 69–70). Herts, England: Turret‐Wheatland Ltd.

[vms3344-bib-0027] Mateos, G. G. , Jiménez‐Moreno, E. , Serrano, M. P. , & Lázaro, R. P. (2012). Poultry response to high levels of dietary fiber sources varying in physical and chemical characteristics. The Journal of Applied Poultry Research, 21, 156–174. 10.3382/japr.2011-00477

[vms3344-bib-0028] McEllhiney, R. R. (1994). Determining and expressing particle size Feed manufacture technology IV (pp. 545–547). Arlington, VA: AFIA Inc.

[vms3344-bib-0029] Moran, E. T. , & Bilgil, S. F. (1992). Colonization from oral salmonella after transient cooping of broilers is reduced as confinement lengthens and when feeds contained a wood sugar pellet binder. Poultry Science, 71S, 47(Abstract).

[vms3344-bib-0030] Moran, E. T. , & Conner, D. E. (1992). Reduction in pH of cecal contents with broiler chicks given probiotic and soluble complex carbohydrates supplemented to the starting feed. Poultry Science, 71S, 167(Abstract).

[vms3344-bib-0055] National Research Council, NRC . (1994). Nutrient requirements of poultry (9th revised ed.). Washington, DC: National Academy Press.

[vms3344-bib-0031] Ohkawa, H. , Ohishi, N. , & Yagi, K. (1979). Assay of lipid peroxides in animal tissues by thiobarbituric acid reaction. Analytical Biochemistry, 95, 351–358. 36810 10.1016/0003-2697(79)90738-3

[vms3344-bib-0032] Ouyang, X. , Qiu, X. , & Chen, P. (2006). Physiochemical characterization of calcium lignosulfonate—A potentiallyuseful water reducer. Colloids Surf A: Physicochemical and Engineering Aspects, 283, 489–497.

[vms3344-bib-0033] Proudfoot, F. G. , & DeWitt, W. F. (1976). The effect of the pellet binder "Lignosol FG" on the chicken's digestive system and general performance. Poultry Science, 55, 629–631. 10.3382/ps.0550629 935019

[vms3344-bib-0035] Pure Lignin Environmental Technology . (2009). Lingin. Retrieved from http://purelignin.com/lignin. Accessed August, 2013.

[vms3344-bib-0036] Safaa, H. M. , Jiménez‐Moreno, E. , Frikha, M. , & Mateos, G. G. (2014). Plasma lipid metabolites and liver lipid components in broilers at 21 days of age in response to dietary different fiber sources. The Egyptian Journal Animal Production, 51, 115–127.

[vms3344-bib-0037] Saki, A. A. , & Alipana, A. (2005). Effect of dietary wheat screening diet on broiler performance, intestinal viscosity and ileal protein digestibility. MSc thesis. Department of Animal Science Bu‐Ali Sina University in Hamedan. Asian network for scientific information, Pakistan. Retrieved from http://www.biomedsearch.com/cite.html?type=bib&doc_num=0001128734.

[vms3344-bib-0038] Saleh, A. A. (2013). Effects of fish oil on the production performances, polyunsaturated fatty acids and cholesterol levels of yolk in hens. Emirates Journal of Food and Agriculture, 25(8), 605–612.

[vms3344-bib-0039] Saleh, A. A. , Ahmed, E. A. M. , & Ebeid, T. A. (2019). The impact of phytoestrogen sources supplementation on reproductive performance, plasma profile, yolk fatty acids and antioxidative status in aged laying hens. Reproduction in Domestic Animals, 54(6), 846–854. 30916364 10.1111/rda.13432

[vms3344-bib-0040] Saleh, A. A. , Ebeid, T. A. , & Abudabos, A. M. (2018). Effect of dietary phytogenics (herbal mixture) supplementation on growth performance, nutrient utilization, antioxidative properties and immune response in broilers. Environmental Science and Pollution Research, 25, 14606–14613. 10.1007/s11356-018-1685-z 29532373

[vms3344-bib-0041] Saleh, A. A. , Hayashi, K. , Ijiri, D. , & Ohtsuka, A. (2015). The influence of dietary supplementation with *Aspergillus awamori* and feeding canola seed on the growth performance and meat quality in male broilers chickens. Journal of Animal Science, 86(3), 305–311. 10.1111/asj.12281 25773115

[vms3344-bib-0042] Saleh, A. A. , Ijiri, D. , & Ohtsuka, A. (2014). Effects of summer shield supplementation on the growth performance, nutrient utilization, and plasma lipid profiles in broiler chickens. Journal of Veterinarni Medicina, 59(11), 536–542.

[vms3344-bib-0043] Saleh, A. A. , Ohtsuka, A. , Yamamoto, M. , & Hayashi, K. (2013). Aspergillus awamori feeding modifies lipid metabolism in rats. BioMed Research International. 2013, 594393 10.1155/2013/594393. 23841078 PMC3694365

[vms3344-bib-0044] Saleh, A. A. , Paray, B. A. , & Dawood, M. A. O. (2020). Olive cake meal and *Bacillus licheniformis* impacted the growth performance, muscle fatty acid content, and health status of broiler chickens. Animals, 10, 695 10.3390/ani10040695 PMC7222747 32316269

[vms3344-bib-0045] Saleh, A. A. , Ragab, M. M. , Ahmed, E. A. M. , Abudabos, A. M. , & Ebeid, T. A. (2018). Effect of dietary zinc‐methionine supplementation on growth performance, nutrient utilization, antioxidative properties and immune response in broiler chickens under high ambient temperature. Journal of Applied Animal Research, 46, 820–827. 10.1080/09712119.2017.1407768

[vms3344-bib-0046] Schell, T. C. , & van Heugten, E. (1998). The effect of pellet quality on grower pigs. Journal of Animal Science, 76(Supplement 1), 185.

[vms3344-bib-0047] Stapelton, P. , Bragg, D. B. , & Biely, J. (1980). The botanical and chemical composition and nutritive value of wheat feed screening. Poultry Science, 59, 333–340.

[vms3344-bib-0057] Tufarelli, V. , Khan, R. U. , & Laudadio, V. (2011). Feeding of wheat middlings in lamb total mixed rations: Effects on growth performance and carcass traits. Animal Feed Science and Technology, 17, 130–135.

[vms3344-bib-0054] Tufarelli, V. , & Laudadio, V. (2011). Effect of wheat middlings‐based total mixed ration on milk production and composition responses of lactating dairy ewes. Journal of Dairy Science, 94, 376–381. 21183048 10.3168/jds.2010-3496

[vms3344-bib-0048] Waldroup, P. W. , Ritchie, S. J. , & Ramsey, B. E. (1982). Effects of lignin sulfonate pellet binder on pellet quality and feeding value for broilers. Feedstuffs, 54(3), 33.

[vms3344-bib-0049] Wamsley, K. G. S. , & Moritz, J. S. (2012). Researching poor pellet quality and maintaining amino acid digestibility in commercial turkey diet feed manufacture. Journal of Applied Poultry Research, 22, 439–446.

[vms3344-bib-0050] Wang, H. , Lichtenstein, A. H. , Lamon‐Fava, S. , & Jacques, P. F. (2006). Association between statin use and serum cholesterol concentrations is modified by whole‐grain consumption. The American Journal of Clinical Nutrition, 100, 1149–1157. 10.3945/ajcn.113.074344 25240077

[vms3344-bib-0051] World Health Organization . (2015). Cardiovascular diseases (CVDs). Facts sheet no 317. Geneva, Switzerland: WHO.

[vms3344-bib-0052] Yang, D. , Qiu, X. , Pang, Y. , & Zhou, M. (2008). Physicochemical properties of calcium lignosulfonate with different molecular weights as dispersant in aqueous suspension. Journal of Dispersion Science and Technology, 29(9), 1296–1303. 10.1080/01932690701866534

[vms3344-bib-0053] Zimonja, O. , Stevnebø, A. , & Svihus, B. (2007). Nutritional value of diets for broiler chickens as affected by fat source, amylose level and diet processing. Canadian Journal of Animal Science, 87, 553–562. 10.4141/CJAS07044

